# Correction: c-Maf restrains T-bet-driven programming of CCR6-negative group 3 innate lymphoid cells

**DOI:** 10.7554/eLife.56774

**Published:** 2020-03-10

**Authors:** Caroline Tizian, Annette Lahmann, Oliver Hölsken, Catalina Cosovanu, Michael Kofoed-Branzk, Frederik Heinrich, Mir-Farzin Mashreghi, Andrey Kruglov, Andreas Diefenbach, Christian Neumann

Tizian C, Lahmann A, Hölsken O, Cosovanu C, Kofoed-Branzk M, Heinrich F, Mashreghi M-F, Kruglov A, Diefenbach A, Neumann C. 2020. c-Maf restrains T-bet-driven programming of CCR6-negative group 3 innate lymphoid cells. *eLife* **9**:e52549. doi: 10.7554/eLife.52549.

Published 10, February 2020

We noticed mistakes in citations (omitted and wrong citations) in the original article. The following citations have been added or changed in the text and reference list:

**#1**

Original text:

In addition, c-Maf may also directly contribute to RORγt expression in ILC3s, as it has been reported for T cells (Tanaka et al., 2014; Zuberbuehler et al., 2019).

Corrected text:

In addition, c-Maf may also directly contribute to RORγt expression in ILC3s, as it has been reported for T cells (Tanaka et al., 2014; Zuberbuehler et al., 2019). Our data supports and extends the findings of a very recent report that was released after completion of this manuscript (Parker et al., 2020).

**#2**

Original text:

Flow cytometry was performed according to previously defined guidelines (Melo-Gonzalez and Hepworth, 2017).

Corrected text:

Flow cytometry was performed according to previously defined guidelines (Cossarizza et al., 2019).

In connection with the changes above, the following citations have been added to the Reference list:

Parker M.E et al., 2020. c-Maf Regulates the Plasticity of Group 3 Innate Lymphoid Cells by Restraining the Type 1 Program. J Exp Med, 217(1).

Cossarizza, A., et al., 2019. Guidelines for the use of flow cytometry and cell sorting in immunological studies (second edition). Eur J Immunol, 49(10): p. 1457-1973.

We apologise for the omission and mistakes of these citations.

In addition to the changes above, the following changes have also been made to the manuscript:

**#3**

Original text:

All animal experiments were in accordance with the ethical standards of the institution or practice at which the studies were conducted and were reviewed and approved by the responsible ethics committees (LAGeSo Berlin, I C 113 – G0172/14).

Corrected text:

All animal experiments were in accordance with the ethical standards of the institution or practice at which the studies were conducted and were reviewed and approved by the responsible ethics committees of Germany (LAGeSo Berlin, I C 113 – G0172/14) and Russia.

We also added the previously omitted funding information for Andrey Kruglov:

Russian Science Foundation grant # 17-74-20059

- Andrey Kruglov

The article has been corrected accordingly.

